# Characterizing MRI features of rectal cancers with different KRAS status

**DOI:** 10.1186/s12885-019-6341-6

**Published:** 2019-11-14

**Authors:** Yanyan Xu, Qiaoyu Xu, Yanhui Ma, Jianghui Duan, Haibo Zhang, Tongxi Liu, Lu Li, Hongliang Sun, Kaining Shi, Sheng Xie, Wu Wang

**Affiliations:** 1 Department of Radiology, China-Japan Friendship Hospital, No.2 Yinghua East Street, Chaoyang District, Beijing, 100029 People’s Republic of China; 2 Philips Healthcare, Beijing, 100001 People’s Republic of China

**Keywords:** Rectal cancer, Magnetic resonance imaging, Texture, KRAS mutation

## Abstract

**Background:**

To investigate whether MRI findings, including texture analysis, can differentiate KRAS mutation status in rectal cancer.

**Methods:**

Totally, 158 patients with pathologically proved rectal cancers and preoperative pelvic MRI examinations were enrolled. Patients were stratified into two groups: KRAS wild-type group (KRAS^wt^ group) and KRAS mutation group (KRAS^mt^ group) according to genomic DNA extraction analysis. MRI findings of rectal cancers (including texture features) and relevant clinical characteristics were statistically evaluated to identify the differences between the two groups. The independent samples t test or Mann-Whitney U test were used for continuous variables. The differences of the remaining categorical polytomous variables were analyzed using the Chi-square test or Fisher exact test. A receiver operating characteristic (ROC) curve analysis was performed to evaluate the discriminatory power of MRI features. The area under the ROC curve (AUC) and the optimal cut-off values were calculated using histopathology diagnosis as a reference; meanwhile, sensitivity and specificity were determined.

**Results:**

Mean values of six texture parameters (Mean, Variance, Skewness, Entropy, gray-level nonuniformity, run-length nonuniformity) were significantly higher in KRAS^mt^ group compared to KRAS^wt^ group (*p* < 0.0001, respectively). The AUC values of texture features ranged from 0.703~0.813. In addition, higher T stage and lower ADC values were observed in the KRAS^mt^ group compared to KRAS^wt^ group (t = 7.086, *p* = 0.029; t = − 2.708, *p* = 0.008).

**Conclusion:**

The MRI findings of rectal cancer, especially texture features, showed an encouraging value for identifying KRAS status.

## Background

Colorectal cancer (CRC) is one of the major causes of cancer-related mortality with over 1 million new cases diagnosed worldwide each year [1, 2]. It is viewed as a heterogeneous disorder due to its molecular features and relevant subtypes, and can be divided into five molecular subtypes correlated to tumor morphological features with different DNA microsatellite instability status and CpG island methylator phenotype [1]. Notably, KRAS mutation is closely linked to villous change and dysplasia [2]. Adenocarcinoma with KRAS mutation that is considered a subgroup of CRC show a negative treatment response to epidermal growth factor receptor (EGFR)-targeted antibodies [3]. Furthermore, KRAS mutation is an established biomarker in clinical practice for CRC and is associated with distant metastasis [4], and poorer survival in CRC [5–7]. Approximately 30–40% CRCs have KRAS mutation, while rectal cancer accounts for 30–35% among CRC [8, 9]. The pre-operative neoadjuvant therapy including anti-EGFR chemotherapy has shown robust value in the management of rectal cancer [3]. Therefore, it is important to select suitable patients who could benefit from aggressive multimodality approaches and to tailor individual treatments against the disease.

Currently, information pertaining to the KRAS status can only be gathered from the biopsy samples or postoperative specimens. Furthermore, the limitations of histological evaluation of KRAS status, such as the variability in the tissue sample and the poor DNA quality in sample results, can lead to discordance between biopsy material and final operative results [10]. Thus, efficient identification of KRAS status in patients with rectal cancer using non-invasively method would be of great clinical interest.

On the other hand, different molecular subtypes correlate with various discriminating morphological features [1]. Various MR imaging modules [11–16] (i.e. diffusion-weighted MR imaging [DWI], magnetic resonance spectroscopy [MRS], arterial spin labelling [ASL]) and advanced analysis for routine MR imaging [17–21] have been introduced in the oncologic field to evaluate tumoral biological characteristics and predict KRAS status. Nevertheless, the radiologic features of rectal cancer with KRAS mutation have not yet been fully described. Texture analysis is a noninvasive method used for assessment of the intra-tumoral heterogeneity not perceptible by human eye, which has a promising value in predicting therapy response, survival and discriminating different stages in rectal cancer [22–24]. However, to date, there have been no studies to assess whether texture analysis of MRI can be used as an imaging biomarker for KRAS status in rectal cancer.

Hence, the main objects of the present study were to 1) retrospectively analyze the differences of radiologic features in rectal cancer with different KRAS status; 2) investigate whether texture features extracted from T2 weighted image scan differentiate KRAS mutation status in rectal cancers.

## Methods

### Patients and tissue samples

This retrospective study was approved by the institutional review board of Institute of Clinical Medicine, China-Japan Friendship Hospital (No. 2015–012), and written informed consent was waived. A total of 220 patients underwent rectal resection for adenocarcinoma with complete clinical data and preoperative pelvic MR examination (including T2WI-high resolution sequence) between June 2013 and September 2015. Exclusion criteria included: i) pre-examination neoadjuvant chemoradiotherapy (*n* = 45) or unidentified herbal medicine therapy (*n* = 5); ii) poor image quality [heavy intestinal peristalsis artifacts (*n* = 10), too small lesions (diameter < 5 mm) or lesions difficult to identify on DWI images (*n* = 2)]. Finally, the group included in the study comprised 158 patients (106 men, 52 women) with a mean age of (60.66 ± 13.38) years (range 26–87 years). Among the 158 subjects, the data of 45 subjects were previously analyzed with a different objective for other research [18].

Surgical pathology results from all patients were analyzed by a pathologist with 6 years’ experience in gastrointestinal pathological diagnosis. Genomic DNA was extracted from formalin fixed paraffin-embedded (FFPE) tissue using QLAamp DNA FFPE Tissue kit (Qiagen, Germany), and KRAS mutations were examined by amplification refractory mutation system (ARMS) method.

### Patient preparation and imaging protocol

Patients were on a low-residue diet before the exam and fasted on the day of the exam. Intramuscular injection of 10 mg anisodamine hydrochloride was given to each patient to inhibit the intestine peristalsis some 10 min before MRI examination. Pelvis MR scanning was implemented on a 3 T whole-body scanner (Ingenia, Philips Medical Systems, Best, the Netherlands) with gradient strength 45mT/m and gradient slew rate 200mT/m/ms, using a 16-channel anterior torso dS coil and a 16-channel posterior table dS coil. 2D sagittal and coronal T2W TSE sequences were performed with following parameters: TR 3761 ms, TE 110 ms, FOV 24 × 24 cm, slice thickness 3 mm with 0.3 mm gap, acquisition matrix 336 × 252, NSA 3. Oblique axial T2W-high resolution sequence was planned perpendicularly to the bowel with tumor: TR 3865 ms, TE 100 ms, FOV 14 × 14 cm, slice thickness 3 mm with 0.3 mm gap, acquisition matrix 232 × 228. Oblique axial diffusion weighted imaging (DWI) scan perpendicularly to the tumor was implemented using a single-shot echo planar imaging with following parameters: TE/TR 76/6000 ms, FOV 20 × 30 cm, slice thickness 5 mm with 0.2 mm gap, acquisition matrix 292 × 304, NSA 6, 2 b values (0,1000s/mm^2^).

### Image analysis

All the data was transmitted to picture archiving and communication system (PACS) and Philips post-processing workstation. Two radiologists (with 11 and 7 years in gastrointestinal imaging), who were blinded to all clinical information, independently measured and recorded the following tumor features: tumor type, location, length, morphologic features, circumferential extent, T staging and the maximal extramural depth (MEMD) of tumor, N staging, circumferential resection margin (CRM), extramural vascular invasion (EMVI), ADC values, textural features. However, they were aware that the study subjects were patients with rectal cancers. For continuous variables, an average value of two observers’ measurement was selected. For categorical variables, the diagnosis was determined after renegotiation by two observers if any interobserver discrepancies occurred.

#### Tumor type

According to the signal intensity of rectal cancers on T2WI [25], the hyperintensity was defined as a signal intensity that was similar to or brighter than the perirectal fat. Each observer quantitatively evaluated hyperintense volume in the tumor and determined the type of tumor as “mucinous” or “non-mucinous” according to the same criteria used for pathologic diagnosis (at least≥50% of the mucin pool occupying the tumor mass [26].

#### Tumor location and length

Tumor location, as well as tumor length were primarily evaluated on sagittal T2-weighted images. Axial and coronal T2-weighted images were used secondarily when required. The rectum was generally divided into three parts according to the anatomic distance from the anal verge: the upper third (> 10 cm), middle third (5-10 cm), lower third(< 5 cm). The anal verge was defined as the end of the anal canal [27]. The distance between the lower margin of rectal lesion and anal verge were measured by drawing along the midline of rectal lumen in a zigzag pattern [28]. Tumor length was also measured along the intestinal lumen in a zigzag pattern.

#### Morphologic criteria/tumor shape

**Fig. 1:**
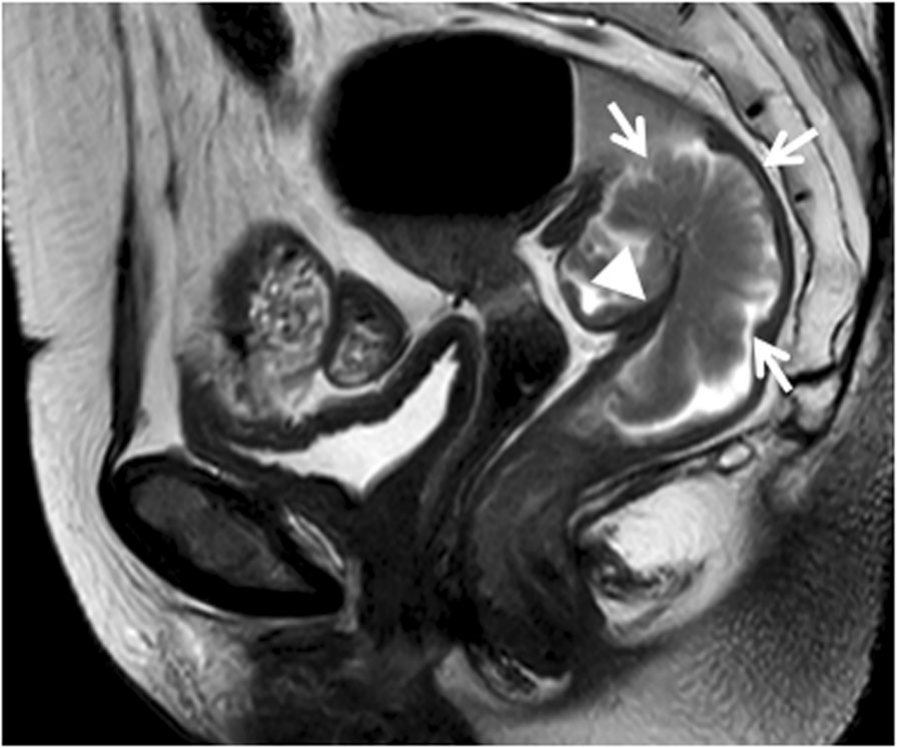
Sagittal T2-weighted imaging of a rectal cancer (intermediate signal intensity) presenting as polypoid mass (arrows) protruded into lumen with distinct intestinal wall (arrow head). Result of the postoperative pathology confirmed that the tumor invaded the submucosa without extending into muscularis propria

**Fig. 2:**
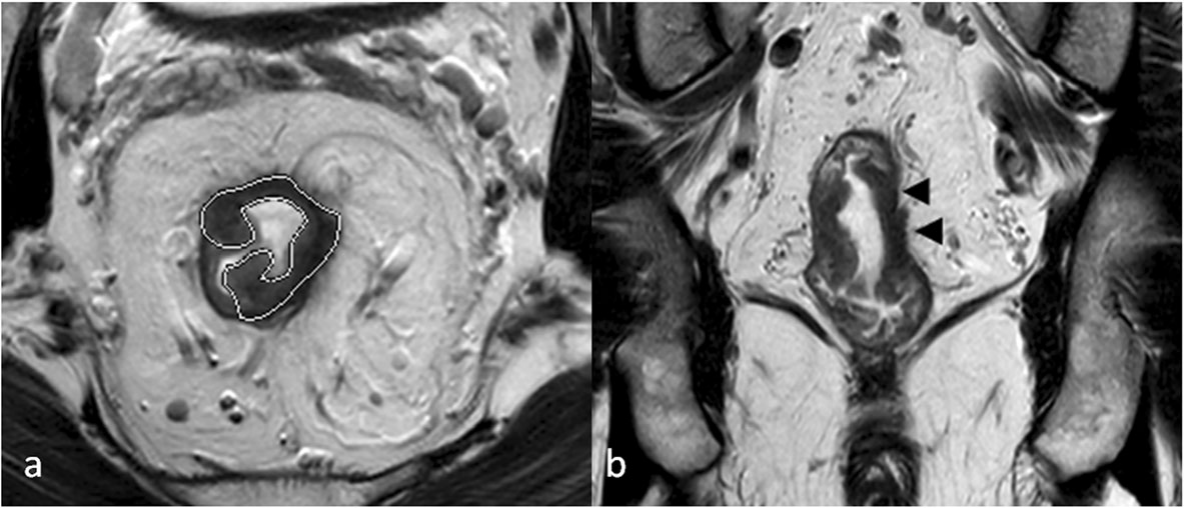
T2-weighted imaging of a rectal cancer (low to intermediate signal intensity) presenting as ulceroinfiltrative mass (**a**, oblique axial, outline indicates tumor region) mainly extended along the intestinal wall with ambiguous muscularis propria (**b**, coronal, arrow head). Final pathologic results demonstrated that tumor invaded through muscularis propria to perirectal tissues

**Fig. 3:**
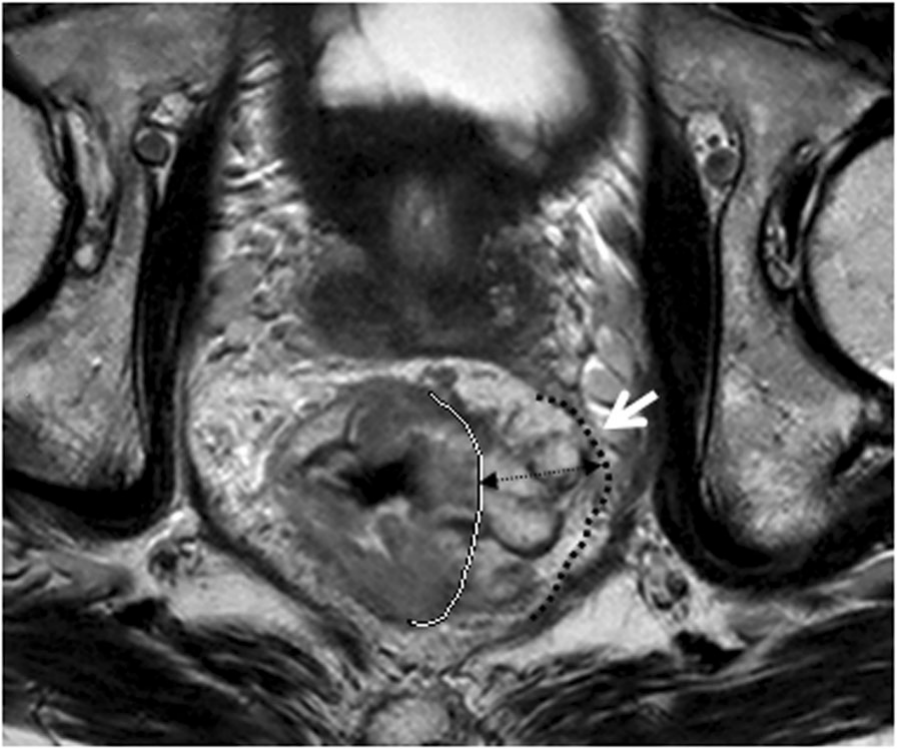
Oblique axial T2-weighted imaging of a rectal mucinous adenocarcinoma (intermediate to high signal intensity) presenting as bulky mass showed significant tumor infiltration beyond the muscularis propria; the maximal extramural depth (MEMD, double-headed arrow) was over 10 mm. Meanwhile, the invasive border of rectal mass bordering the mesorectal fascia (white arrow) which leaded to a CRM of 0 mm. White line = muscularis propria border. Black dashed line = the mesorectal fascia

Tumor shapes were classified [27] as (a) intraluminal polypoid lesion (without abutting pericolorectal tissues) (Fig. 1); (b) ulcerofungating/ulceroinfiltrative mass (Fig. 2); (c) bulky (Fig. 3). If the tumor showed growth tendency of protruding mass into colorectal lumen or limited thickening-wall with a sharp margin from the adjacent normal intestinal wall, without breaching the outer margin, it was considered as the intraluminal polypoid lesion. If the tumor demonstrated wall-thickening grow tendency with abutting pericolorectal tissue, and MEMD < 10 mm, it was considered as the ulcerofungating/ulceroinfiltrative mass. If the tumor showed exophytic growth tendency with disproportionately expanding component outside the imaginary line of the main tumor (MEMD≥10 mm), and the outer diameter of the tumor-bearing segment was larger than that of the adjacent normal colorectal segment, then it was considered a bulky mass.

#### Circumferential extent

Axis bowel (clock face) was divided into quarters, C1: tumor extent≤1/4 bowel circumference; C2: tumor extent> 1/4 bowel circumference and ≤ 1/2 bowel circumference; C3: tumor extent > 1/2 bowel circumference and ≤ 3/4 bowel circumference; C4: tumor extent > 3/4 bowel circumference.

#### Tumor and node staging

Primary tumor and lymph node stage were observed on MRI [29] by using the Tumor-Node-Metastasis (TNM) staging system. Meanwhile, the MEMD of tumor was recorded, and T3 sub-stages were then classified [30] according to different MEMD. T3 sub-stage: T3a: MEMD < 1 mm beyond muscularis propria; T3b: MEMD ≥1-5 mm beyond muscularis propria; T3c: MEMD > 5-15 mm beyond muscularis propria; T3d: MEMD > 15 mm beyond muscularis propria. Nodes with irregular borders, mixed signal intensity, or both were suspected for metastasis, and presence of one to three suspicious nodes was defined as stage N1 and presence of four or more as stage N2.

#### CRM

The potential positive margin was defined as rectal tumor spread within 1 mm of the mesorectal fascia (Fig. 3), that occurred due to tumor deposits, tumor extramural extent, EMVI, or suspicious lymph nodes [30].

#### EMVI

**Fig. 4:**
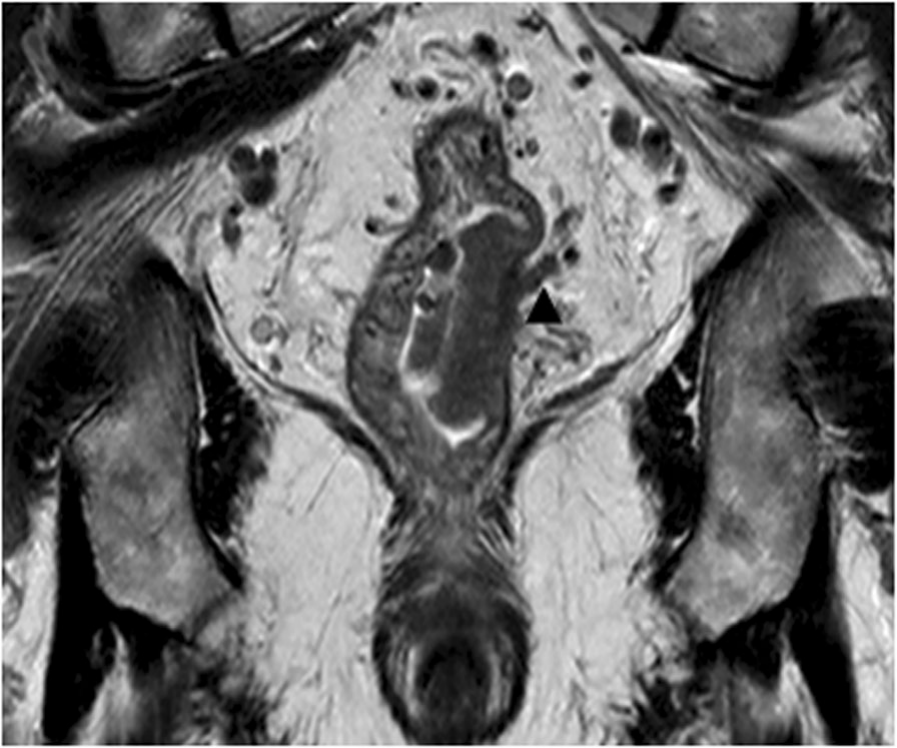
Extramural vascular invasion (EMVI) involvement. Coronal T2-weighted imaging showing focal expansion of the small perirectal vessel with intermediate signal intensity (black arrow head)

EMVI was defined as the presence of rectal tumor cells within blood vessels located beyond the muscularis propria in the mesorectal fat [30]. The following clues for EMVI (Fig. 4) were (a) vessel expanded by tumor, having irregular contour; (b) presence of tumor signal intensity within vascular structure.

#### ADC evaluation

Images of diffusion-weighted (DW) sequence were transferred to the Extended Workspace 4.1 (Philips Medical Systems, Best, Netherlands). Regions of interest (ROIs) were manually drawn to cover the entire tumor area on the axial slices containing all available tumor areas, which appeared as high signal on the DW images, avoiding the gas in the bowel and other anatomy structures.

#### Textural features

For each tumor, consecutive three axial T2W images (encompassing the tumor maximum cross-section as the middle slice) were conducted for textural analysis by using MaZda, version 4.6 (P.M. Szczypiński, Institute of Electronics, Technical University of Lodz, Poland). Freehand ROIs were delineated with the tumor contour on axial images avoiding the inclusion of intestinal gas, liquid and anatomical structures. Although contouring was performed using T2WI images, the observers looked at DW images, when available, to most accurately place the ROI.

Prior to analysis, MR image intensities were normalized between the range [μ-3σ, μ + 3σ], where μ was the mean value of gray levels inside the region of interest and σ denoted the standard deviation. Gray levels between [μ-3σ] and [μ + 3σ] were then decimated to 64 Gy levels. This normalization procedure has been shown to minimize inter-scanner effects in MRI feature analysis [31]. Given that this analysis produced much more features than positive cases in the study, only first-and second-order texture features (three features) were selected for further analysis to avoid overfitting [32, 33]. Totally, 25 parameters, which are listed in Table 1, were extracted for each ROI on each slice. Run-length matrix (RLM) parameters were calculated four times for each ROI (vertical, horizontal, 45°, 135°) and grey-level co-occurrence matrix (GLCM) parameters were calculated 20 times for each ROI at a variety of pixel offsets. For the comparison of textural features between tumors with different KRAS status, the mean value of gray-level histogram, RLM and GLCM parameters were used for each ROI, providing in total 25 parameters for analysis. Then, three parameters derived from gray-level histogram (*Mean*, *Variance*, *Skewness*), one parameter from gray-level co-occurrence matrix (GLCM) (*Entropy*) and two parameters from RLM (*gray-level nonuniformity* [GLNU],*run-length nonuniformity* [RLNU]) were extracted for each of the three slices based on the probability of classification error and the average correlation coefficients (POE + ACC) [34]. The detailed description of the calculated texture parameters was provided by Haralick et al. [35].

**Table 1:** Summary of parameters belonging to first- and second-order texture features^33-35^

Texture feature	Histogram (*n*=9)	Run-length matrix (*n*=5)	Grey-level co-occurrence matrix (*n*=11)
Level/Order	First order	Second order	Second order
Description	Histogram where x-axis represents pixel/voxel gray level and y-axis represents frequency of occurrence	Adjacent or consecutive pixels/voxels of a single gray level in a given direction	How often pairs of pixels with specific values in a specified spatial range occur in an image
Parameters	Mean	Short run-length emphasis	Angular second moment
	standard deviation	Long run-length emphasis	Contrast
	skewness	Run-length non-uniformity	Correlation
	Kurtosis	Grey-level non-uniformity	Sum of squares
	Perc.1%	Fraction of image in runs	Inverse difference moment
	Perc.10%		Sum average
	Perc.50%		Sum variance
	Perc.90%		Sum entropy
	Perc.99%		Entropy
			Difference variance
			Difference entropy

The selected feature sets were evaluated using the computer program B11, which is part of the MaZda software package. Artificial neural network (ANN) classifier [34] was employed for investigating the ability of texture feature sets to distinguish between rectal cancers with different KRAS status. The classification results were arbitrarily divided into several levels according to the misclassification rates: excellent (misclassification rates≤10%), good (10% < misclassification rates≤20%), moderate (20% < misclassification rates≤30%), fair (30% < misclassification rates≤40%), and poor (misclassification rates≥40%) [36].

### Statistical analysis

The statistical analysis was performed by SPSS (SPSS 17.0 for Windows, SPSS, Chicago, IL). The Kolmogorov-Smirnov test for normality was performed on continuous variables and the graphical spread of the data was visually inspected. Descriptive statistics were shown as mean ± standard deviation (SD) or median ± interquartile range (IQR) for continuous variables, and as frequency and percentage for categorical variables. Interobserver agreement for continuous variables (ADC values, tumor length, MEMD, textural parameters) was evaluated using the intra-class correlation coefficient (ICC), and for categorical variables using Kappa of agreement. The Kappa value was interpreted as follows: < 0, poor agreement; 0 to 0.20, slight agreement; 0.21 to 0.40, fair agreement; 0.41 to 0.60, moderate agreement; 0.61 to 0.80, substantial agreement; and > 0.80, almost perfect agreement.

Patients were stratified into two groups: KRAS wild-type group (KRAS^wt^ group) and KRAS mutation group (KRAS^mt^ group) according to genomic DNA extraction and analysis. Mann-Whitney U test was used to compare variables (MEMD, texture features) with abnormal distribution for differentiation of rectal cancers with different KRAS status. The independent samples *t* test was used to compare other continuous variables (including ADC values, length and patients’ age) between KRAS^wt^ and KRAS^mt^ group. Then, the differences among the other categorical variables were analyzed using the chi-square test or Fisher exact test. A receiver operating characteristic (ROC) curve analysis was performed to evaluate the discriminatory power of MRI features including ADC values, tumor shape, T stage and textural features in differentiating tumor KRAS mutation. The area under the curve (AUC) and optimal cutoff values were calculated, as well as the corresponding sensitivity and specificity. *P*<0.05 indicated a statistically significant difference.

## Results

### Patient characteristics

Of 158 patients (mean age, 60.66 ± 13.38), 143 (90.51%) had cancers detected according to symptoms such as abdominal pain, hematochezia, changes in bowel habits and diarrhea, 10 (6.33%) had screen-detected cancers, and the cancers in the remaining 5 patients (3.16%) were discovered during the examination for other diseases. According to final pathological results, 98 (62.03%) had KRAS^wt^ and 60 (37.97%) had KRAS^mt^ type cancer. MRI features and patients’ clinical characteristics in KRAS^wt^ group and KRAS^mt^ group are shown in Table 2.

**Table 2:** MRI features and clinical characteristics of patients with rectal cancer(n=158)

Factors	Total (No./values)	KRAS Status		*P*-value
		Wild-type (*n*=98)	Mutant (*n*=60)	
Age	60.66±13.38	60.42±12.89	61.07±14.26	0.388
Gender				0.769
Male	106	66(67.35%)	40(66.67%)	
Female	52	32(32.65%)	20(33.33%)	
ADC (×10^-3^mm^2^/ms )	1.22±0.39	1.37±0.37	1.15±0.38	0.008^*^
Texture features				
Mean	66.47±14.55	62.66±10.53	73.34±18.38	<0.0001
Variance	289.19±118.96	267.65±122.51	334.39±94.27	<0.0001
Skewness	0.54±0.67	0.43±0.53	0.73±0.42	<0.0001
Entropy	1.89±0.23	1.80±0.19	1.97±0.14	<0.0001
RLUN	178.38±65.19	159.87±53.38	208.12±69.14	<0.0001
GLUN	7.53±3.44	6.55±2.83	9.26±3.33	<0.0001
Tumor location				
Upper Rectum	48	33(33.67%)	15(25.00%)	0.095
Middle Rectum	72	47(47.96%)	25(41.67%)	
Lower Rectum	38	18(18.37%)	20(33.33%)	
Tumor shape				0.022
Intraluminal polypoid mass	40	31(31.63%)	9(15.00%)	
Ulcerofungating/Ulceroinfiltrative mass	95	57(58.16%)	38(63.33%)	
Bulky	23	10(10.20%)	13(21.67%)	
Tumor length(cm)	4.12±1.68	4.04±1.62	4.25±1.78	0.446^*^
Tumor type				0.556
Mucinous adenocarcinoma	23	13(13.27%)	10(16.67%)	
Non-mucinous adenocarcinoma	135	85(86.73%)	50(83.33%)	
Cirumferential extent				0.872^**^
C1	6	4(4.08%)	2(3.33%)	
C2	56	36(36.73%)	20(33.33%)	
C3	56	34(34.69%)	22(36.67%)	
C4	40	24(24.49%)	16(26.67%)	
Radiologic T stage				0.029
T1-2	49	35(35.71%)	14(23.33%)	
T3	94	58(59.18%)	36(60.00%)	
T4	15	5(5.10%)	10(16.67%)	
T3 substage				0.041
T3a	23	19(32.76%)	4(11.11%)	
T3b	36	23(39.66%)	13(36.11%)	
T3c	23	11(18.97%)	12(33.33%)	
T3d	12	5(8.62%)	7(19.44%)	
MEMD(cm)	0.30±0.60	0.30±0.60	0.50±0.60	0.028^*^
N stage				0.754
N0	74	48(48.98%)	26(43.33%)	
N1	51	31(31.63%)	20(33.33%)	
N2	23	19(19.39%)	14(23.33%)	
EMVI				0.664
Positive	34	20(20.41%)	14(23.33%)	
Negative	124	78(79.59%)	46(76.67%)	
CRM				0.337
Positive	38	28(28.57%)	13(21.67%)	
Negative	120	70(71.43%)	47(78.33%)	

*Abbreviations*: *ADC* apparent diffusion coefficient, *MEMD* the maximal extramural depth of tumour; *EMVI* extramural vascular invasion, *CRM* circumferential resection margin, *RLNU* run-length nonuniformity, *GLNU* grey-level nonuniformity

*independent samples t test, data is mean ± standard deviation;Δ Mann-Whitney U test, data is data is median ± interquartile range. **Considering limited patients’ numbers in subgroups of circumferential extent, reclassification was adopted as follows: C1-2, C3, and C4, and *P* value was the result of new categorization

### Quantitative textural analysis and ADC

Mean values of six texture features were significantly different in rectal cancers with different KRAS status (*p* < 0.0001). In addition, good classification results (error of 12.7%) were obtained with ANN classifier. Lower ADC values were observed in the KRAS^mt^ group compared to the KRAS^wt^ group (*t* = − 2.708, *p* = 0.008). The observed results are listed in Table 2.

### Conventional imaging analysis

With regard to tumor shape, the shape distribution between the two groups was quite different (*x*^2^=7.591, *p* = 0.022), with higher incidences of bulky (21.67%) and less intraluminal polypoid mass (15.00%) in the KRAS^mt^ group compared to (10.20 and 31.63%, respectively) KRAS^wt^ group, respectively. In addition, higher T stage was observed more frequently in the KRAS^mt^ group compared to the KRAS^wt^ group (*x*^2^=7.086, *p* = 0.029). Moreover, the mean MEMD in the KRAS^mt^ group was significantly larger than in the KRAS^wt^ group (Z = -2.202, *p* = 0.028), and relevant T3 sub-stage distribution in two groups showed a similar trend (*x*^2^=8.240, *p* = 0.041).

Although rectal cancers with KRAS mutation were mainly located in the middle-low part of rectum and had an extent of over 3/4 bowel circumference, there was no statistical difference between the KRAS^mt^ group and the KRAS^wt^ group (*p* = 0.095 and 0.872, respectively). Other imaging features including length, N staging, EMVI, CRM also demonstrated no significant difference between the two groups. Moreover, the incidence of mucinous adenocarcinomas in the KRAS^mt^ group was higher than in the KRAS^wt^ group. Yet, no significant difference was demonstrated (*x*^2^=0.346, *p* = 0.556) between the two groups. The observed results are listed in Table 2.

### ROC analysis

**Fig. 5:**
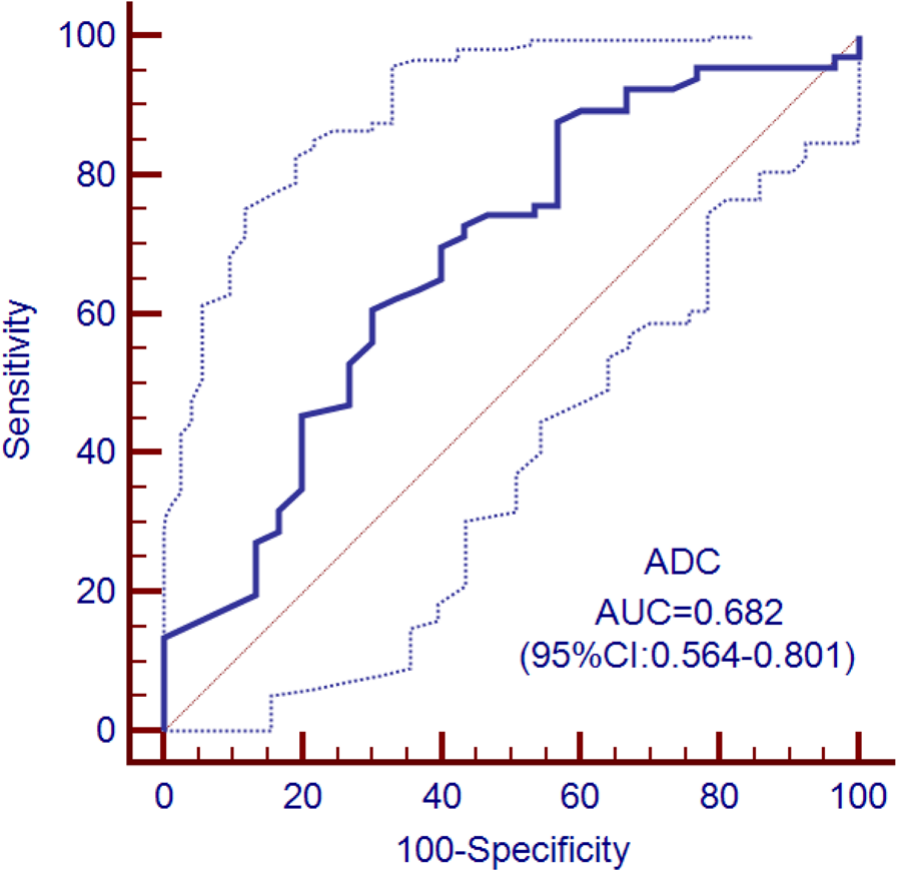
Receiver operating characteristic (ROC) curves (solid line) and 95% confidence bounds (dotted lines) for ADC values in differentiating KRAS mutation status in rectal cancer

**Fig. 6:**
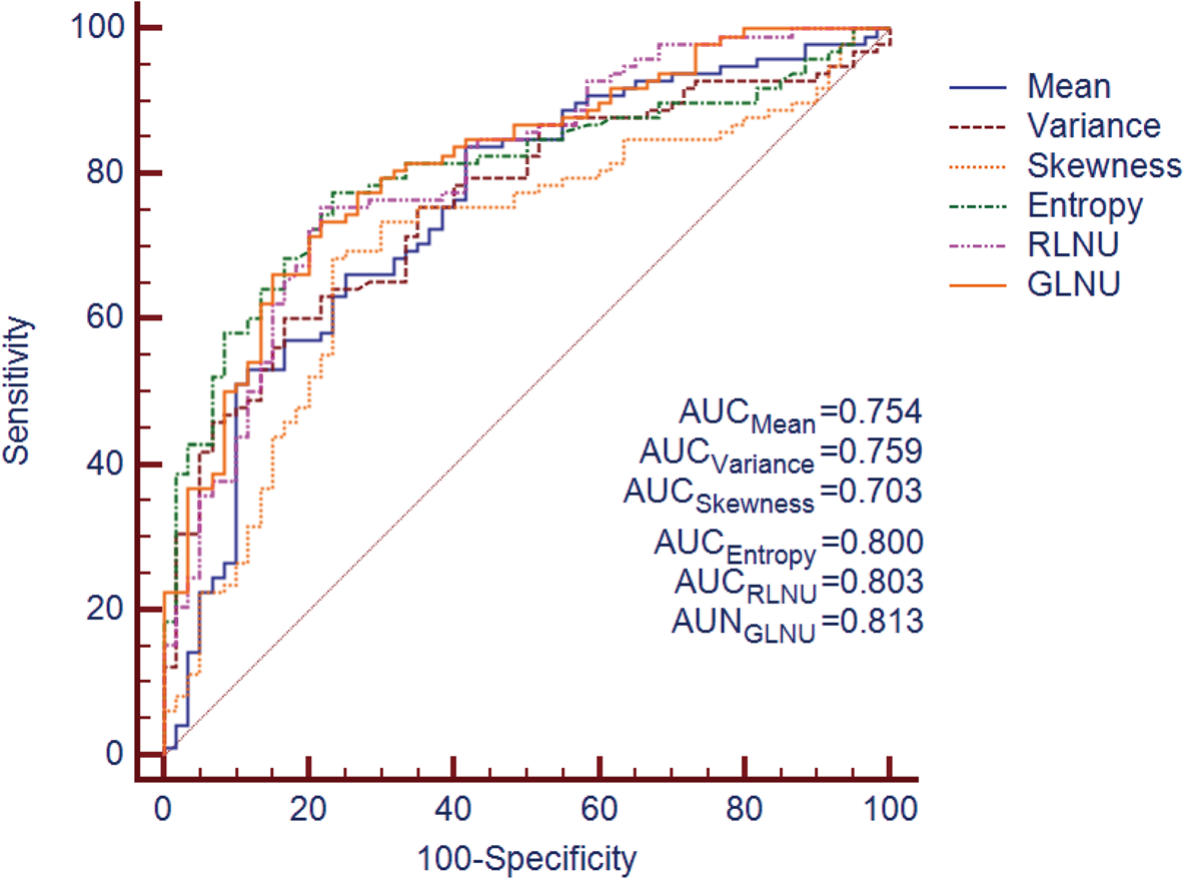
Receiver operating characteristic (ROC) curves corresponding to T2W images derived quantitative texture features for differentiating KRAS mutation status in rectal cancer

The ROC curve of the ADC values is shown in Fig. 5. The AUC of ADC values was 0.682 (95%CI: 0.564~0.801); at a cutoff value of 1.145 × 10^− 3^ mm^2^/s, the sensitivity and specificity were 66.67, 62.12%, respectively. The ROC curve of the quantitative texture values is shown in Fig. 6. According to ROC curve, textural features: *Mean*, *Variance*, *Skewness*, *Entropy*, GLUN and RLUN values showed diagnostic significance with the AUC values of 0.754, 0.759, 0.703, 0.800, 0.802 and 0.813, respectively. The optimal cutoff values for the above features and their relevant sensitivity, and specificity are listed in Table 3.

**Table 3:** Receiver operator characteristics of textural parameters for predicting KRAS status

Feature	AUC	SE(AUC)	95%CI(AUC)	Criterion	Se(%)	Sp(%)
Mean	0.754	0.040	0.674~0.833	>72.072	58.33%	83.67%
Variance	0.759	0.038	0.684~0.834	>281.700	83.33%	60.20%
Skewness	0.703	0.043	0.618~0.787	>0.554	80.00%	69.39%
Entropy	0.800	0.035	0.731~0.870	>1.893	76.67%	78.57%
RLNU	0.802	0.036	0.731~0.872	>186.350	78.33%	76.53%
GLNU	0.813	0.034	0.746~0.880	>7.846	78.33%	74.49%

*Abbreviation*: *AUC* area under the curve, *SE* standard error, *Se* sensitivity, *Sp* specificity, *RLNU* run-length nonuniformity, *GLNU* gray-level nonuniformity

### Interobserver agreement

Relatively good to excellent interobserver agreement was obtained for continuous variables ADC values, tumor length, MEMD, textural features with ICC values ranging from 0.719 to 0.9487, 0.9838 to 0.9963, 0.9643 to 0.9918, and 0.6379 to 0.8159, respectively. The interobserver agreement for categorical variables measured by the Kappa value ranged from 0.729 to 1.0. EMVI had a substantial agreement (Kappa value, 0.729), while the remaining MRI features showed almost perfect agreement (Kappa value > 0.8) (Table 4).

**Table 4:** Inter-observer agreement for variables

Inter-observer agreement for variables			
Variable Type	Variable	Kappa value/ICC	95%CI
Categorical	Tumor shape	0.919	0.863-0.974
	circumferential	0.979	0.965-1.0
	T stage	0.935	0.883-0.986
	N stage	0.940	0.898-0.981
	Tumor type	0.804	0.663-0.945
	EMVI	0.729	0.599-0.857
	CRM	0.812	0.694-0.930
Continuous	ADC	0.8542	0.7190-0.9487
	Tumor length	0.9885	0.9838-0.9963
	MEMD	0.9843	0.9643-0.9918
	Mean	0.7448	0.6663-0.8069
	Variance	0.7571	0.6818-0.8159
	Skewness	0.7402	0.6607-0.8032
	Entropy	0.7452	0.6670-0.8072
	GLNU	0.7239	0.6379-0.7916
	RLNU	0.7539	0.6776-0.8141

Inter-observer agreement of categorical variables was evaluated by Kappa or weighted Kappa value, while inter-observer agreement of continuous ones was evaluated by ICC

## Discussion

In the present study, we found that 1) the textural analysis based on T2 weighted images had robust value in differentiating KRAS status in rectal cancer; 2) rectal cancers with KRAS mutation showed lower ADC value and manifested as ulcerofungating/ ulceroinfiltrative mass or had bulky shape, behaving more aggressive to surrounding tissue with larger MEMD and higher T stage. To our knowledge, this study is the first that explored the potential of textural analysis for predicting KRAS status in rectal cancer based on MR images.

Although textural features are inconsistent for variable software vendors, the focus key in texture analysis has been on assessing heterogeneity in tumor images [37]. Each texture feature measures a particular property of the arrangement of pixels within ROIs. Theoretically, a number of these features are correlated with intra-tumor heterogeneity attributed to various factors including necrosis, hypoxia, angiogenesis, hemorrhage, even genetic variations [37–41] For example, *Variance* is negatively associated with angiogenesis in CRCs without KRAS mutant, while positive association has been demonstrated between *Skewness* and angiogenesis in CRCs with KRAS mutant [41]. *Entropy* derived from GLCM measures the disorder of an image [35]. If the image is heterogeneous, many of the elements in the co-occurrence matrix will have very small values, thus implying a very large entropy [42]. In the present study, rectal cancer with KRAS mutation had higher *Entropy* values compared to the KRAS^wt^ group (*p* < 0.0001). In other words, rectal cancer with KRAS mutation had higher intrinsic heterogeneity than KRAS wild-type cancers did; this intrinsic heterogeneity in KARS mutation should be addressed more in detail by further research.

Encouraging results on texture analysis for differentiating benign and malignant lymph nodes, identifying T stage and predicting outcome after chemoradiotherapy in rectal cancer have been reported by previous studies using different imaging modalities including CT, MR, and ultrasound [22–24, 43, 44]. In this study, we performed the texture analysis of rectal cancer using T2 weighted images from MR, which is the gold-standard imaging technique for preoperative staging and is also the standard routine for patients with rectal cancer at our hospital. Furthermore, MRI could reduce the impact of image noise on biological heterogeneity with higher contrast resolution and contrast-to-noise ratio compared with CT [24].

As mentioned above, the lower ADC value observed in rectal cancers with KRAS mutation may suggest an unfavorable tumor profile. Recent studies have revealed that low ADC values are associated with poorly differentiated tumors and high tumor stages in rectal cancers [45, 46] It is well known that ADC value is inversely correlated with the cellularity and positively correlated with necrosis and cystic changes in tissues. Hence, lower ADC value might reflect less necrosis, higher cellular density, and higher vascularization, suggesting the aggressiveness of the tumor profile [47]. Furthermore, it has been reported that lung metastasis is more likely to develop in CRCs with KRAS mutation than in KRAS wild-type [48]. These findings are indirectly consistent with our results.

In the present study, higher incidences of bulky CRCs were observed in the KRAS^mt^ group compared to the KRAS^wt^ group. Kim et al [27] have shown a higher incidence of bulky CRCs in the poorly differentiated CRCs than in the well- or moderately differentiated CRCs, and poor differentiation is associated with high risk of postoperative relapse in stage II CRCs [49]. Thus, it is proposed that bulky CRCs are more likely to have a poor prognosis. Actually, according to classification criteria [27], bulky tumors had exophytic growth tendency with MEMD> 10 mm in our study. Cho and colleagues [49] have reported that significantly higher 3-year recurrence rate after surgical treatment is observed in rectal cancers with MEMD> 10 mm than in tumors with MEMD ≤10 mm, which supported our hypothesis. Consequently, it is easy to understand the relatively higher incidence of bulky CRCs in the KRAS^mt^ group.

In daily practice, the biopsy is still the routine way to get tumour mutant status before treatment. Considering the spatial and temporal intra-tumour molecular heterogeneity, the results for biopsy samples are yet to be consistent [11–13, 50]. Recently, a large prospective study [50] showed that the concordance ratio between paired biopsy and resection specimens was 82% for KRAS status. In the current study, although the best sensitivity (*Variance*) and specificity (*Mean*) of the texture features were both over 83% in the study, the sensitivity and specificity of GLNU, which harboured the best diagnostic significance (AUC = 0.813), were both lower than 80%. These findings suggest that the textural analysis can potentially provide promising MRI biomarkers for KRAS status, however, the sensitivity of it still needs to be improved in further studies.

There are some limitations in the current study. First, texture features were selected using POE + ACC algorithms in combination with ANN classifiers, and merely six features were extracted for further analysis in this study. Considering that a large number of features could be generated by MaZda software and that limited subjects were included in the study selective bias may exist and further studies are required. Second, due to the complexity of the technique and a high number of parameters, high variability in data acquisition could be introduced in the MRI scan, and in theory could affect the reproducibility of the final results [24]. However, the differences in texture features extracted from MR images from different scanners seem to have only a weak impact on the results of tissue discrimination [34]. Third, with regard to the genomic results, our data were restricted to the KRAS mutations located in codons 12 and 13. Nevertheless, since condons 12 and 13 KRAS mutations represent the majority of RAS mutations in CRC, our results provide a reasonable representation for tumors with RAS mutation in some degree. Fourth, considering the potential discrepancy between pre-treatment biopsy and final pathology [11–13], only the outcome from final surgical specimen were enrolled in the study. Fifth, this was a single-center study with a limited sample size, which may be the reason why only moderate predictive value of MRI features for identifying KRAS status has been observed. Further work with a larger sample size may lead to more statistically significant results.

## Conclusion

Overall, our preliminary results demonstrate that MRI features, including quantitative texture analysis derived from T2 weighted images, have the potential to differentiate the KRAS status in rectal cancers. The additional texture features may provide reference information for characterizing KRAS status with the expected impact on management of individualized diagnosis and treatment of rectal cancer.

## Abbreviations

ADC: Apparent diffusion coefficient
ANN: Artificial neural network
ARMS: Amplification refractory mutation system
AUC: The area under the ROC curve
CRC: Colorectal cancer
CRM: Circumferential resection margin
DWI: Diffusion weighted imaging
EGFR: Epidermal growth factor receptor
EMVI: Extramural vascular invasion
FFPE: Formalin fixed paraffin-embedded
FOV: Field of view
GLCM: Grey-level co-occurrence matrix
GLNU: Gray-level nonuniformity
ICC: Intra-class correlation coefficient
IQR: Interquartile range
KRAS: Kirsten rat sarcoma viral oncogene homolog
MEMD: Maximal extramural depth
MRI: Magnetic resonance imaging
PACS: Picture archiving and communication system
RLM: Run-length matrix
RLNU: Run-length nonuniformity
ROC: Receiver operating characteristic
ROI: Region of interest
SD: Standard deviation
TE: Echo time
TNM: Tumor Node Metastasis
TR: Repetition time
TSE: Turbo spin echo

## References

[1] Coppedè F, Lopomo A, Spisni R, Migliore L (2014). Genetic and epigenetic biomarkers for diagnosis, prognosis and treatment of colorectal cancer. World J Gastroenterol.

[2] Jass JR (2007). Classification of colorectal cancer based on correlation of clinical, morphological and molecular features. Histopathology.

[3] Yadamsuren EA, Nagy S, Pajor L, Lacza A, Bogner B (2012). Characteristics of advanced- and non advanced sporadic polypoid colorectal adenomas: correlation to KRAS mutations. Pathol Oncol Res.

[4] Misale S, Di Nicolantonio F, Sartore-Bianchi A, Siena S, Bardelli A (2014). Resistance to anti-EGFR therapy in colorectal cancer: from heterogeneity to convergent evolution. Cancer Discov.

[5] Zhu K, Yan H, Wang R, Zhu H, Meng X, Xu X (2014). Mutations of KRAS and PIK3CA as independent predictors of distant metastases in colorectal cancer. Med Oncol.

[6] Qiu LX, Mao C, Zhang J, Zhu XD, Liao RY, Xue K (2010). Predictive and prognostic value of KRAS mutations in metastatic colorectal cancer patients treated with cetuximab: a meta-analysis of 22 studies. Eur J Cancer.

[7] Guo TA, Wu YC, Tan C, Jin YT, Sheng WQ, Cai SJ (2019). Clinicopathologic features and prognostic value of KRAS, NRAS and BRAF mutations and DNA mismatch repair status: a single-center retrospective study of 1,834 Chinese patients with stage I-IV colorectal cancer. Int J Cancer.

[8] Boeckx N, Peeters M, Van Camp G, Pauwels P, Op de Beeck K, Deschoolmeester V (2015). Prognostic and predictive value of RAS gene mutations in colorectal Cancer: moving beyond KRAS exon 2. Drugs.

[9] Hardiman KM (2018). Update on sporadic colorectal Cancer genetics. Clin Colon Rectal Surg.

[10] Andreyev HJ, Norman AR, Cunningham D, Oates J, Dix BR, Iacopetta BJ (2001). Kirsten ras mutations in patients with colorectal cancer: the ‘RASCAL II’ study. Br J Cancer.

[11] Rönnow CF, Uedo N, Stenfors I, Toth E, Thorlacius H (2019). Forceps biopsies are not reliable in the workup of large colorectal lesions referred for endoscopic resection: should they be abandoned?. Dis Colon Rectum.

[12] Nelson AC, Boone J, Cartwright D, Thyagarajan B, Kincaid R, Lambert AP (2018). Optimal detection of clinically relevant mutations in colorectal carcinoma: sample pooling overcomes intra-tumoral heterogeneity. Mod Pathol.

[13] Jeantet M, Tougeron D, Tachon G, Cortes U, Archambaut C, Fromont G (2016). High Intra- and Inter-Tumoral Heterogeneity of RAS Mutations in Colorectal Cancer. Int J Mol Sci.

[14] Abdel Razek AAK (2018). Routine and advanced diffusion imaging modules of the salivary glands. Neuroimaging Clin N Am.

[15] Abdel Razek AAK (2018). Diffusion tensor imaging in differentiation of residual head and neck squamous cell carcinoma from post-radiation changes. Magn Reson Imaging.

[16] Abdel Razek AA, Samir S, Ashmalla GA (2017). Characterization of parotid tumors with dynamic susceptibility contrast perfusion-weighted magnetic resonance imaging and diffusion-weighted Mr imaging. J Comput Assist Tomogr.

[17] Razek AA, Nada N (2016). Correlation of choline/Creatine and apparent diffusion coefficient values with the prognostic parameters of head and neck squamous cell carcinoma. NMR Biomed.

[18] Xu Y, Xu Q, Sun H, Liu T, Shi K, Wang W (2018). Could IVIM and ADC help in predicting the KRAS status in patients with rectal cancer?. Eur Radiol.

[19] Abdel Razek AAK, Talaat M, El-Serougy L, Gaballa G, Abdelsalam M (2019). Clinical applications of arterial spin labeling in brain tumors. J Comput Assist Tomogr.

[20] Yeo DM, Oh SN, Choi MH, Lee SH, Lee MA, Jung SE (2018). Histogram analysis of perfusion parameters from dynamic contrast-enhanced MR imaging with tumor characteristics and therapeutic response in locally advanced rectal Cancer. Biomed Res Int.

[21] Meng X, Xia W, Xie P, Zhang R, Li W, Wang M (2019). Preoperative radiomic signature based on multiparametric magnetic resonance imaging for noninvasive evaluation of biological characteristics in rectal cancer. Eur Radiol.

[22] Liu L, Liu Y, Xu L, Li Z, Lv H, Dong N (2017). Application of texture analysis based on apparent diffusion coefficient maps in discriminating different stages of rectal cancer. J Magn Reson Imaging.

[23] Jalil O, Afaq A, Ganeshan B, Patel UB, Boone D, Endozo R (2017). Magnetic resonance based texture parameters as potential imaging biomarkers for predicting long-term survival in locally advanced rectal cancer treated by chemoradiotherapy. Color Dis.

[24] De Cecco CN, Ganeshan B, Ciolina M, Rengo M, Meinel FG, Musio D (2015). Texture analysis as imaging biomarker of tumoral response to neoadjuvant chemoradiotherapy in rectal cancer patients studied with 3-T magnetic resonance. Investig Radiol.

[25] Hussain SM, Outwater EK, Siegelman ES (1999). Mucinous versus nonmucinous rectal carcinomas: differentiation with MR imaging. Radiology.

[26] Luo C, Cen S, Ding G, Wu W (2019). Mucinous colorectal adenocarcinoma: clinical pathology and treatment options. Cancer Commun.

[27] Kim JE, Lee JM, Baek JH, Moon SK, Kim SH, Han JK (2015). Differentiation of poorly differentiated colorectal adenocarcinomas from well- or moderately differentiated colorectal adenocarcinomas at contrast-enhanced multidetector CT. Abdom Imaging.

[28] Han NY, Kim MJ, Park BJ, Sung DJ (2014). Location of rectal cancer as determined using rectal magnetic resonance imaging, and its relationship with pulmonary metastasis. Turk J Gastroenterol.

[29] Taylor FG, Swift RI, Blomqvist L, Brown G (2008). A systematic approach to the interpretation of preoperative staging MRI for rectal cancer. AJR Am J Roentgenol.

[30] Nougaret S, Reinhold C, Mikhael HW, Rouanet P, Bibeau F, Brown G (2013). The use of MR imaging in treatment planning for patients with rectal carcinoma: have you checked the “DISTANCE”?. Radiology.

[31] Collewet G, Strzelecki M, Mariette F (2004). Influence of MRI acquisition protocols and image intensity normalization methods on texture classification. Magn Reson Imaging.

[32] Szczypiński PM, Strzelecki M, Materka A, Klepaczko A (2009). MaZda--a software package for image texture analysis. Comput Methods Prog Biomed.

[33] Lubner MG, Smith AD, Sandrasegaran K, Sahani DV, Pickhardt PJ (2017). CT texture analysis: definitions, applications, biologic correlates, and challenges. Radiographics.

[34] Mayerhoefer ME, Breitenseher MJ, Kramer J, Aigner N, Hofmann S, Materka A (2005). Texture analysis for tissue discrimination on T1-weighted MR images of the knee joint in a multicenter study: transferability of texture features and comparison of feature selection methods and classifiers. J Magn Reson Imaging.

[35] Haralick R, Shanmugam K, Dinstein I (1973). Textural features for image classification. IEEE Trans Syst Man Cybern.

[36] Yan L, Liu Z, Wang G, Huang Y, Liu Y, Yu Y, Liang C (2015). Angiomyolipoma with minimal fat: differentiation from clear cell renal cell carcinoma and papillary renal cell carcinoma by texture analysis on CT images. Acad Radiol.

[37] Miles KA, Ganeshan B, Hayball MP (2013). CT texture analysis using the filtration-histogram method: what do the measurements mean?. Cancer Imaging.

[38] Gerashchenko TS, Denisov EV, Litviakov NV, Zavyalova MV, Vtorushin SV, Tsyganov MM (2013). Intratumor heterogeneity: nature and biological significance. Biochemistry (Mosc).

[39] Nelson DA, Tan TT, Rabson AB, Anderson D, Degenhardt K, White E (2004). Hypoxia and defective apoptosis drive genomic instability and tumorigenesis. Genes Dev.

[40] Ng F, Ganeshan B, Kozarski R, Miles KA, Goh V (2013). Assessment of primary colorectal cancer heterogeneity by using whole-tumor texture analysis: contrast-enhanced CT texture as a biomarker of 5-year survival. Radiology.

[41] Ganeshan B, Ziauddin X, Goh V, Rodriguez-Justo M, Engledow A, Taylor S (2012). Quantitative imaging biomarkers from PET-CT as potential correlates for angiogenesis and hypoxia in colorectal cancer.

[42] Andrea B, Flavio P (1995). An investigation of the textural characteristics associated with gray level co-occurrence matrix statistical parameters. IEEE Trans Geoscience Remote Sensing.

[43] Chen SJ, Lin CH, Chang CY (2012). Characterizing the major sonographic textural difference between metastatic and common benign lymph nodes using support vector machine with histopathologic correlation. Clin Imaging.

[44] Bayanati H, E Thornhill R, Souza CA (2015). Quantitative CT texture and shape analysis: can it differentiate benign and malignant mediastinal lymph nodes in patients with primary lung cancer?. Eur Radiol.

[45] Curvo-Semedo L, Lambregts DM, Maas M, Beets GL, Caseiro-Alves F, Beets-Tan RG (2012). Diffusion-weighted MRI in rectal cancer: apparent diffusion coefficient as a potential noninvasive marker of tumor aggressiveness. J Magn Reson Imaging.

[46] Sun Y, Tong T, Cai S, Bi R, Xin C, Gu Y (2014). Apparent diffusion coefficient (ADC) value: a potential imaging biomarker that reflects the biological features of rectal cancer. PLoS One.

[47] Xiao-ping Y, Jing H, Fei-ping L, Yin H, Qiang L, Lanlan W (2016). Intravoxel incoherent motion MRI for predicting early response to induction chemotherapy and chemoradiotherapy in patients with nasopharyngeal carcinoma. J Magn Reson Imaging.

[48] Pereira AA, Rego JF, Morris V, Overman MJ, Eng C, Garrett CR (2015). Association between KRAS mutation and lung metastasis in advanced colorectal cancer. Br J Cancer.

[49] Cho SH, Kim SH, Bae JH, Jang YJ, Kim HJ, Lee D (2014). Prognostic stratification by extramural depth of tumor invasion of primary rectal cancer based on the Radiological Society of North America proposal. AJR Am J Roentgenol.

[50] Sclafani F, Wilson SH, Cunningham D, et al. Analysis of KRAS, NRAS, BRAF, PIK3CA and TP53 mutations in a large prospective series of locally advanced rectal cancer patients. Int J Cancer. 2019. 10.1002/ijc.32507 [Epub ahead of print]. 10.1002/ijc.32507 31199501

